# Differential expression of syndecans and glypicans in chronically inflamed synovium

**DOI:** 10.1136/ard.2006.063875

**Published:** 2007-06-01

**Authors:** A M Patterson, A Cartwright, G David, O Fitzgerald, B Bresnihan, B A Ashton, J Middleton

**Affiliations:** 1 Leopold Muller Arthritis Research Centre, Institute for Science and Technology in Medicine, Medical School, Keele University at Robert Jones and Agnes Hunt Orthopaedic Hospital, Oswestry, Shropshire, UK; 2 Center for Human Genetics, University of Leuven, Campus Gasthuisberg, Leuven, Belgium; 3 University Department of Rheumatology, St Vincents Hospital, Dublin, Ireland

## Abstract

**Background:**

Membrane-bound heparan sulphate proteoglycans (HSPGs) act as co-receptors and presenters of cytokines and are involved in cell–matrix and cell–cell adhesion.

**Aim:**

To investigate which HSPGs are expressed in knee joint synovia from patients with different forms of arthritis and normal individuals.

**Methods:**

Synovial samples were obtained from patients with early rheumatoid arthritis (n = 8), longstanding rheumatoid arthritis (n = 13), psoriatic arthritis (n = 7), osteoarthritis (n = 6) and normal joints (n = 12). Expression of syndecan-1, -2, -3 and -4 and glypican-1, -3 and -4 was analysed by immunohistochemistry and dual label immunofluorescence.

**Results:**

The expression of HSPGs in chronically inflamed synovium exhibited a differential distribution. Syndecan-1 was present in the mononuclear infiltrates of synovia from patients with rheumatoid and psoriatic arthritis where it was expressed by plasma cells. Syndecan-2 was present mainly in blood vessels where it occurred on endothelial cells, pericytes and smooth muscle cells. Syndecan-3 stained intensely in endothelial cells but also occurred in sublining macrophages and the lining layer. Glypican-4 occurred in the lining layer and blood vessels. Increased expression of these HSPGs was apparent in rheumatoid and psoriatic compared to osteoarthritic and normal synovia. Little or no staining for syndecan-4, glypican-1 and glypican-3 was seen in all samples.

**Discussion:**

Selected HSPGs, such as syndecan-1, -2 and -3 and glypican-4, could play a part in the pathophysiology of arthritis, such as the migration and retention of leukocytes and angiogenesis in the chronically inflamed synovium.

Proteoglycans are composed of glycosaminoglycan (GAG) chains, such as heparan sulphate, chondroitin sulphate, keratan sulphate or dermatan sulphate, covalently attached to a core protein. Two major classes of proteoglycans contain heparan sulphate chains: syndecans, which have a transmembrane domain in their core proteins, and glypicans, which are attached to the cell membrane by glycosylphosphatidylinositol (GPI)-anchors.^1^ ^2^ In the basement membrane perlecan is the major component that bears heparan sulphate.

To date, four syndecans and six glypicans have been identified. Syndecans are the major source of cell surface heparan sulphate. They are expressed in a cell-, tissue- and development-specific manner.^1^ Syndecan-1 and -4 have been shown in endothelial cells,^3^ however, syndecan-1 is mainly expressed on epithelial cells with syndecan-4 expression on many cell types. Changes in their expression occur during embryogenesis, wound healing and carcinogenesis.^4^^–^6 Although syndecan-2 has been identified as an endothelial heparan sulphate proteoglycan (HSPG),^7^ expression within tissues has also been shown on fibroblasts, for example, in skin and periodontium,^8^ with expression also occurring on carcinoma cells.^9^ Syndecan-3 was first identified on neuronal cells and has been associated with the generation of cerebellar fibrillar plaques in Alzheimer disease.^10^ It is also an HSPG of the musculoskeletal system.^11^^–^14 Glypicans are widely expressed in embryonic and adult tissues such as ovary, intestine and central nervous system, and are involved in growth factor signalling.^15^ They play a role in tissue growth, regeneration and cancer.

Rheumatoid arthritis (RA) is characterised by chronic inflammation of the synovium of the joints, resulting in stiffness, pain and—as the disease progresses—erosion of the joint tissues and deformities.^16^ Psoriatic arthritis (PsA) resembles RA in being an inflammatory disease leading to joint destruction, but differs from RA in several ways including the distribution of affected joints, the presence of skin lesions and enthesopathy, and the absence of rheumatoid factor, characteristic rheumatoid erosions and periarticular osteopoenia on radiographs. The exact pathogenesis of RA and PsA is largely unknown, but it is clear that a number of factors may be involved either individually or in combination. During RA, characteristic histopathological changes occur; the synovial lining layer undergoes thickening and hypertrophy, and in the sublining leukocytes such as monocytes, T cells and B cells migrate into the tissue where they accumulate.^17^

There is increasing evidence that HSPGs are involved in inflammation.^18^ Using animal knockout models and isolated cells, syndecan-1 and syndecan-4 have been shown to be involved in regulating inflammatory responses,^19^ binding chemokines^20^ ^21^ and forming chemokine gradients.^22^ ^23^ The chemokine CXCL8 has been shown to bind to syndecan-2 in cultured human umbilical vein endothelial cells^24^ and we recently showed the induction of a CXCL8 binding site on syndecan-3 in the endothelial cells of the RA synovium.^14^ However, little is known about the expression of syndecans and glypicans by the various cell types of the chronically inflamed synovium, although other proteoglycans bearing GAGs such as dermatan sulphate have been identified in this tissue.^25^ HSPGs are of interest as they are co-receptors for cytokines (eg, fibroblast growth factor), presenters of chemokines and are involved in cell–matrix and cell–cell adhesion.^26^^–^30 Hence they are likely candidates involved in several pathomechanisms in chronically inflamed synovia, such as angiogenesis and the migration and retention of leukocytes. Therefore, this study aimed to compare the expression patterns of syndecan-1, -2, -3, and -4, and glypican-1, -3 and -4 in the RA, PsA and normal synovium.

## MATERIALS AND METHODS

### Tissue samples

All samples of synovia were obtained, with informed consent, from the suprapatellar pouch and medial gutter from knee joints. Clinical and demographic details are given in table 1. For early RA, synovia were taken by arthroscopic needle biopsy from patients diagnosed with RA according to the American College of Rheumatology (ACR) criteria and had a mean disease duration of <12 months. RA synovia were also taken at total knee replacement or synovectomies from patients who fulfilled the ACR criteria and had a disease duration >5 years. Synovia were obtained by arthroscopic biopsy of knees of patients with PsA. Osteoarthritic (OA) synovia were obtained at total knee replacement operations. Non-RA synovia, which were histologically normal, were taken arthroscopically from patients who had knee joint symptoms for suspected joint damage. Tissue was frozen in liquid nitrogen-cooled isopentane and stored in the vapour phase of liquid nitrogen. Normal human skin samples were kindly provided by Dr E Johnston and Dr C Mangham, Department of Pathology, University of Birmingham, UK.

**Table 1:** Clinical and demographic details of patients

Patient	Gender	Age	Diagnosis	Duration (years)	ESR	RF (titre)	Histology score	Medication
1	M	52	Normal (meniscal tear)	1.2	ND	ND	0	None
2	F	53	Normal (articular cartilage degeneration)	0.25	ND	ND	1	None
3	M	57	Normal (articular cartilage degeneration)	1	ND	ND	0	None
4	F	57	Normal (meniscal tear)	1.5	ND	ND	0	None
5	M	58	Normal (meniscal tear and cartilage degeneration)	0.5	ND	ND	0	None
6	F	41	Normal (articular cartilage degeneration)	0.5	ND	ND	0	None
7	M	38	Normal (articular cartilage damage)	1	ND	ND	0	None
8	F	55	Normal (meniscal and articular cartilage damage)	2	ND	Negative	0	None
9	F	29	Normal (articular cartilage degeneration)	11	<10	Negative	0	None
10	M	28	Normal (meniscal tear)	1	ND	ND	0	None
11	M	44	Normal (meniscal tear)	15	ND	ND	0	NSAID
12	M	55	Normal (articular cartilage damage)	>1	ND	ND	0	None
1	ND	ND	ERA	1.5	14	Positive (640)	3	NSAID
2	ND	ND	ERA	0.25	23	Positive (80)	1	NSAID
3	ND	ND	ERA	0.6	ND	ND	1	ND
4	ND	ND	ERA	0.25	51	Positive (640)	2	NSAID
5	ND	ND	ERA	0.5	44	Positive (640)	2	None
6	ND	ND	ERA	0.3	34	Positive (170)	1	None
7	ND	ND	ERA	1	48	Positive (160)	2	None
8	ND	ND	ERA	0.1	12	Positive (640)	3	NSAID
1	F	48	RA	18	24	Positive (400)	3	MTX, NSAID
2	F	47	RA	15	18	Negative	1	St, NSAID, analgesic
3	F	62	RA	26	64	Positive (>1280)	2	Sulphasalazine, NSAID
4	F	45	RA	20	71	Positive (640)	3	Sulphasalazine, azathioprine, NSAID
5	M	73	RA	15	59	Positive(1280)	2	d-Penicillamine, NSAID, analgesic
6	F	43	RA	5	23	Positive (640)	2	MTX
7	F	52	RA	20	30	Positive (1280)	3	MTX, Hydroxychloroquine, NSAID, st, gold
8	F	67	RA	7	105	Positive (160)	3	Steroid, NSAID
9	F	63	RA	8	100	Positive (320)	3	MTX, st, analgesic
10	F	62	RA	38	100	Positive (640)	3	MTX, st
11	F	55	RA	21	68	Positive (1280)	2	NSAID
13	F	68	RA	28	70	Positive (800)	2	Analgesic
1	M	42	OA	2	ND	Negative	1	None
2	F	61	OA	>4	ND	ND	2	Analgesic
3	F	72	OA	>5	ND	ND	1	NSAID, analgesic
4	F	33	OA	20	ND	Negative	1	NSAID
5	M	78	OA	15	ND	ND	1	NSAID
6	F	83	OA	13	ND	ND	2	NSAID
1	M	22	PsA	8	11	Negative	1	Aulin
2	F	40	PsA	<1	28	Negative	2	NSAID, st, tar pomeda, reductil
3	F	60	PsA	13	4	Negative	2	Betnovate, Lipitor
4	F	50	PsA	<1	5	Negative	0	DMARD
5	M	35	PsA	2	11	Negative	2	NSAID, Losec, analgesic
6	F	55	PsA	28	10	Negative	3	St, MTX
7	F	27	PsA	20	24	Negative	3	NSAID, st

DMARD, disease-modifying antirheumatic drug; ERA, early rheumatoid arthritis; MTX, methotrexate; ND, not determined; NSAID, non-steroidal anti-inflammatory drug; OA, osteoarthritis; PsA, psoriatic arthritis; RA longstanding rheumatoid arthritis; RF, rheumatoid factor; St, steroid.

### Antibodies

The antibodies used were: anti-syndecan-1 (clone B-B4; Serotec, Oxford, UK); anti-syndecan-2 (mouse IgG1, clone 10H4), anti-syndecan-3 (mouse IgG1 clone 1C7), anti-syndecan-4 (mouse IgG1 clone 8G3), anti-glypican-1 (mouse IgG1 clone S1) (all prepared and supplied by Dr G David, University of Leuven, Belgium), anti-glypican-3 (Santa Cruz Biotechnologies, Santa Cruz, California, USA), anti-glypican-4 (rabbit polyclonal antibody kindly donated by Professor HD Haubeck, Germany), anti-human IgG (rabbit polyclonal, Dako code A 0423; Dako, Glostrup, Denmark), anti-CD20 (Dako clone L26), anti-CD68 (rabbit polyclonal; Santa Cruz Biotech, code sc-9139). The antibodies to the syndecans and glypicans have already been shown to be specific using biochemical approaches (eg, western blotting) and specific staining has been obtained for other human tissues.^31^^–^37

### Immunohistochemistry

The method of Roskams *et al*^38^ was followed, using the anti-syndecan and anti-glypican-1 antibodies, with minor modifications; for glypican-3 the method of Patterson *et al*^14^ for polyclonal antibodies was used with minor modifications, for glypican-4 the polyclonal antibody was diluted in 10% human serum and detected with swine anti-rabbit horse radish peroxidase conjugate diluted in 10% human serum. Briefly, 10 μm thick serial cryostat sections of synovia and skin were dried for 1 h and stored at −80°C until required. Prior to immunohistochemical analysis, slides were left to equilibrate to room temperature for 30 min, fixed in acetone (4°C) for 10 min, air dried, and then sections were rehydrated in phosphate buffered saline (PBS) for 5 min. All primary antibodies were used at 5 μg/ml since initial experiments showed that this concentration gave the optimum specific staining over the range 2.5–10 μg/ml. Antibody binding was detected using DAB staining kit (Vector Labs, Burlingame, California, USA). Sections were counterstained with Mayer Haematoxylin and mounted. In control experiments isotype matched control mouse Ig or rabbit Ig were added instead of primary antibodies, and stained with the DAB kit as above.

### Double label immunofluorescence

For co-localisation of syndecan-1 and IgG, sections were treated with anti-syndecan-1 and anti-IgG (both at 5 μg/ml) added together, washed in PBS and incubated with goat anti-mouse IgG1–fluorescein isothiocyanate (FITC) (1:500 Southern Biotech code 1070-02; Southern Biotech, Birmingham, Alabama, USA) and swine anti-rabbit Ig-biotinylated (1:500 Dako code E0353) containing 10% human serum. The detection of the biotinylated secondary antibody was by using streptavidin-Alexa 594 (1:1000 Molecular Probes code S-11227; Molecular Probes, Eugene, Oregon, USA). For co-localisation of syndecan-1 and CD20, sections were treated with anti-syndecan-1 (5 μg/ml) and anti-CD20 (2 μg/ml), washed in PBS and incubated with goat anti-mouse IgG1–FITC (as above) and goat anti-mouse IgG2b-Texas red (1:100 Southern Biotech code 1090-07) containing 10% human serum. For co-localisation of syndecan-3 and CD68, sections were incubated with anti-syndecan-3 (5 μg/ml) and anti-CD68 (4 μg/ml), washed in PBS and treated with goat anti-mouse IgG-Alexa 488 (1:500 Molecular Probes code A11001) and goat anti-rabbit-Texas red (1:100 Santa Cruz Biotechnology code sc-2780) containing 10% human serum. All sections were washed in PBS, rinsed with water, air dried and mounted. In control experiments, isotype-matched control mouse, goat or rabbit Ig were added instead of primary antibodies, and treated with relevant secondary antibodies as above.

### Analysis

The immunoreactivity of HSPG expression was scored blind by two individuals using the following scoring system that consisted of a combination of staining intensity and number of positive cells: no staining (–), weak (+), strong (++) and very strong (+++) (table 2). A histological scoring system was used to assess the degree of synovial inflammation:^14^ 0 for normal intimal layer and no leukocyte infiltration; 1 for small foci/areas of leukocyte infiltration with two or more synovial lining cells; 2 for synovitis with moderate infiltrations and three or more synovial lining cells; 3 for severe synovitis with widespread infiltration and four or more synovial lining cells (table 1).

## RESULTS

Cryosections of RA, PsA, OA and non-RA synovia, and normal skin were incubated with antibodies to each of syndecans-1 to -4, glypicans-1, –3, and -4 and perlecan.

**Figure 1:**
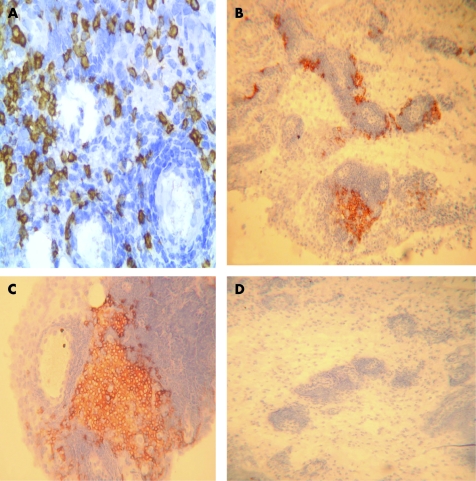
Expression of syndecan-1 in synovia. Sections of human knee synovium were incubated with an antibody to syndecan-1 and processed using immunoperoxidase and diaminobenzidine (brown). Staining is present in the mononuclear infiltrates of patients with early rheumatoid arthritis (A), longstanding rheumatoid arthritis (B) and psoriatic arthritis (C). (D) is a negative control section from the same individual as (B) except the primary antibody was replaced by isotype-matched control IgG1. Original magnification A ×250, B and D ×60, C ×120.

**Figure 2:**
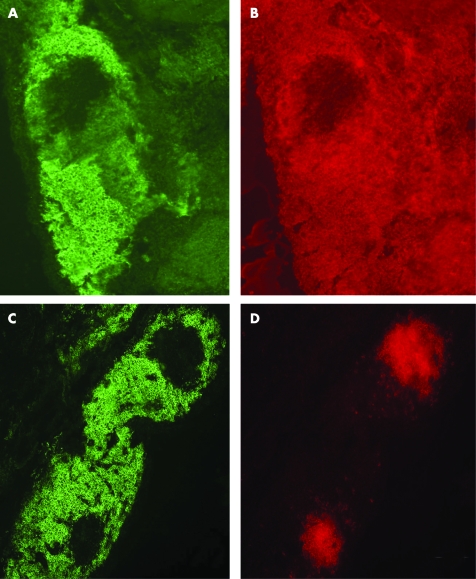
Co-localisation of syndecan-1 with IgG. Sections of synovia from longstanding rheumatoid arthritis were treated with antibodies to syndecan-1 and human IgG and observed by double label immunofluorescence microscopy. Syndecan-1 positive cells (A) co-localise with IgG (B). Lack of co-distribution of syndecan-1 (C) and CD20 (D). Original magnification ×100 (A-D).

Using immunohistochemistry, intense staining for syndecan-1 was seen in the lymphocytic infiltrates of patients with longstanding RA and PsA (fig 1, table 2). Staining was also present in these infiltrates in early RA (fig 1A), but was weak in OA and absent in normal synovia. Using double label immunofluorescence staining, these syndecan-1 positive cells co-localised with IgG suggesting that they were plasma cells (fig 2A,B) and their histological appearance was that of plasma cells. Syndecan-1 and IgG immunoreactivity were negative in normal synovia. The syndecan-1 positive cells did not co-localise with CD20 indicating that they were not immature or mature B cells (fig 2C,D).

**Table 2:** Cellular distribution of heparan sulphate proteoglycans in normal (n = 12), early rheumatoid (n = 8), longstanding rheumatoid (n = 13),osteoarthritis (n = 6) and psoriatic (n = 7) synovium

Tissue	Syndecan-1	Syndecan-2	Syndecan-3	Syndecan-4	Glypican-1	Glypican-3	Glypican-4
Normal							
Lining layer	−	−	+	−	−	−	+
Sublining:							
Fibroblasts	−	−	+	−	−	−	−
Mononuclear infiltrates	−	−	−	−	−	−	−
Macrophages	−	−	+	−	−	−	−
Endothelium	−	+	+	−	−	−	+
Smooth muscle cells	−	+	−	−	−	−	+
Pericytes	−	+	−	−	−	−	+
Early rheumatoid						ND	
Lining layer	−	+	++	+	−		++
Sublining:							
Fibroblasts	−	−	+	−	−		−
Mononuclear infiltrates	++	+	−	−	−		−
Macrophages	−	−	++	−	−		−
Endothelium	−	++	+++	−	−		+
Smooth muscle cells	−	++	−	−	−		+
Pericytes	−	+	−	−	−		+
Longstanding rheumatoid							
Lining layer	−	−	++	−	−	−	++
Sublining:							
Fibroblasts	−	−	+	−	−	−	−
Mononuclear infiltrates	+++	+	−	−	−	−	−
Macrophages	−	−	++	−	−	−	−
Endothelium	−	++	+++	−	−	−	+
Smooth muscle cells	−	++	−	−	−	−	+
Pericytes	−	++	−	−	−	−	+
Osteoarthritis							
Lining layer	−	−	+	−	−	−	+
Sublining:							
Fibroblasts	−	−	+	−	−	−	−
Mononuclear infiltrates	+	−	−	−	−	−	−
Macrophages	−	−	++	−	−	−	−
Endothelium	−	++	++	−	−	−	+
Smooth muscle cells	−	++	−	−	−	−	+
Pericytes	−	+	−	−	−	−	+
Psoriatic							
Lining layer	−	+	++	+	−	−	++
Sublining:							
Fibroblasts	−	−	+	−	−	−	−
Mononuclear infiltrates	+++	+	−	−	−	−	−
Macrophages	−	−	++	−	−	−	−
Endothelium	−	++	+++	−	−	−	+
Smooth muscle cells	−	++	−	−	-	−	+
Pericytes	−	++	−	−	−	−	+

Immunoreactivity of cells: − no staining; + weak staining; ++ strong staining; +++ very strong staining.

ND, not determined.

**Figure 3:**
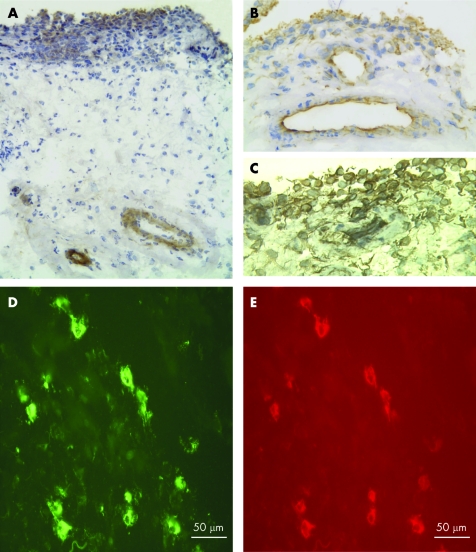
Expression of syndecan-2 and syndecan-3 in synovia. A. Immunohistochemistry showing staining of syndecan-2 in the walls of blood vessels in the sublining and in the lining layer of rheumatoid arthrtitis (RA) synovium. B. Syndecan-3 staining within endothelial cells and the lining layer in early RA. C. Detail of syndecan-3 staining of the lining layer in longstanding RA. D,E. Double label immunofluorescence microscopy with syndecan-3 (D) and CD68 (E) antibodies showing syndecan-3 localising to sublining macrophages in RA. Original magnification ×100 (A), ×200 (B) ×300 (C-E).

Syndecan-2 showed a different distribution compared to syndecan-1. It occurred mainly within the walls of blood vessels (fig 3A) in all samples with strong staining in RA, OA and PsA and weak staining in normal synovia (table 2). The endothelial cells, pericytes and smooth muscle cells were positive. Some additional staining was present in the lining layer of early RA and PsA samples.

Syndecan-3 also localised to blood vessels where it showed a more selective distribution than syndecan-2; intense labelling occurred on endothelial cells but pericytes and smooth muscle cells were negative (fig 3B, table 2). Staining was stronger in the endothelial cells of chronically inflamed synovia from RA and PsA, compared to OA and normal. This is in agreement with our earlier study showing more intense staining of this HSPG in synovial endothelial cells of longstanding RA compared to normal.^14^ Cells within the sublining connective tissue were positive for syndecan-3. These cells were macrophages, as judged by their morphology, and this was confirmed by double label experiments that showed syndecan-3 co-localising with CD68 positive cells in RA and normal synovia (fig 3D,E). CD68 negative cells were also positive for syndecan-3 in the sublining. These cells were identified as being fibroblasts, based on their morphology, whereas lymphocytic infiltrates were negative. The lining layer stained strongly for syndecan-3 in RA and PsA and weakly in OA and normal (fig 3C, table 2).

**Figure 4:**
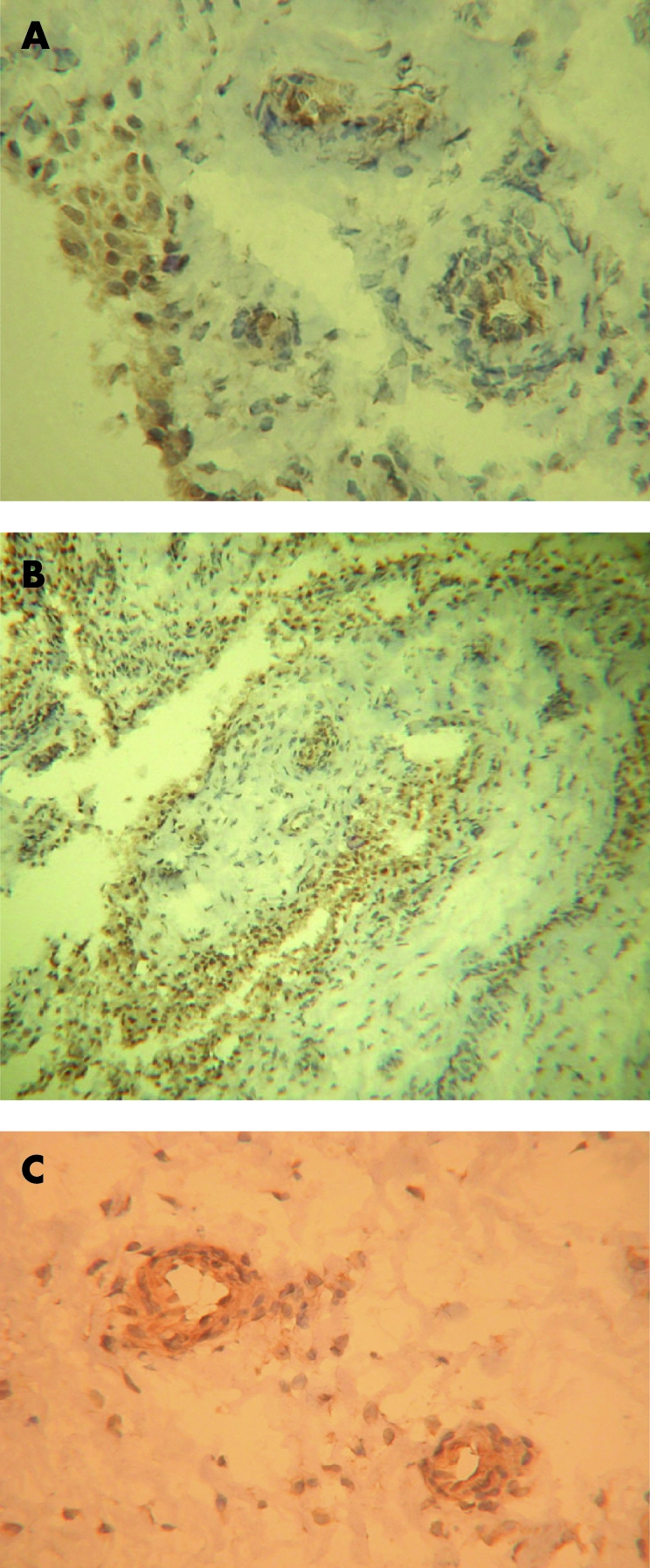
Glypican-4 expression in synovia. A. Staining occurs in the lining layer and the blood vessels in early rheumatoid arthritis (RA) synovium. B. The presence of glypican-4 in the lining layer in longstanding RA. In normal synovium (C), staining occurs in the walls of blood vessels. Original magnifications A and C ×100, B ×50.

In synovia from all patients groups, glypican-4 staining occurred in the blood vessel wall, localising to the endothelium, pericytes and smooth muscle cells (fig 4, table 2). In addition, glypican-4 was demonstrated in the lining layer where strong staining was found in early RA, longstanding RA and PsA. The staining pattern of perlecan was localised to the lining layer, and in the sublining positivity was found in the walls of blood vessels (data not shown). This staining pattern was present in the synovia from all patient groups and is in agreement with a previous study of OA synovium.^39^

**Figure 5:**
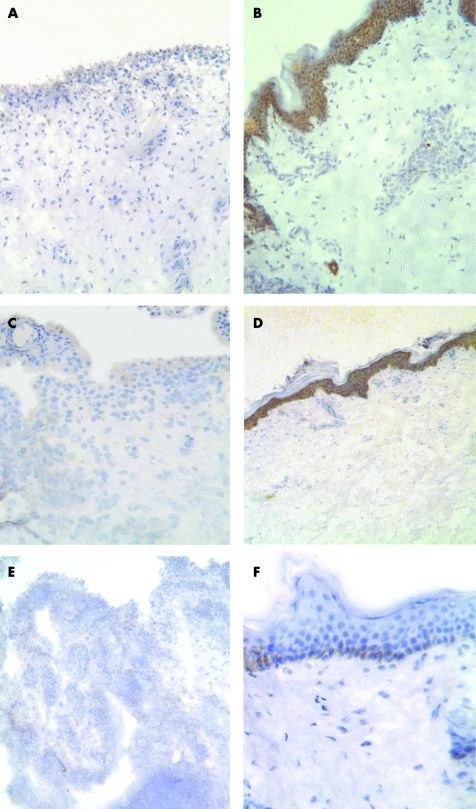
Immunohistochemistry for syndecan-4, glypican-1 and glypican-3 in synovia and skin. Lack of syndecan-4 (A), glypican-1 (C) and glypican-3 (E) staining in rheumatoid arthritis (RA) synovia. However, staining for syndecan-4 (B) and glypican-1 (D) does occur in keratinocytes throughout the epidermis and glypican-3 is present in the basal cell layer of the epidermis. Original magnifications: A and B ×150, C and F ×350, D and E ×60.

Syndecan-4 immunoreactivity was negative in all synovia (fig 5A) apart from some weak staining in the lining layer of early RA and PsA (table 2). Synovial staining for glypican-1 and glypican-3 was negative (fig 5C,E). However, expression of syndecan-4, glypican-1 and glypican-3 was detected in the epidermis (fig 5B,D,F) and blood vessels of human skin, similar to that previously reported,^8^ providing a positive control for these antibodies.

Immunostaining was negative in all experiments in which the primary antibodies to HSPGs were replaced with the same concentrations of control mouse (fig 1D), goat or rabbit Ig.

## DISCUSSION

The results of the present study show that the HSPGs expressed in chronically inflamed synovium show a differential expression pattern. Syndecan-1 was present in the mononuclear infiltrates of synovia from patients with RA and PsA where it occurred in plasma cells. This HSPG has been used as a marker of epithelial cells and plasma cells.^40^^–^44 In the present study it was a selective marker for the latter cell type, which is in agreement with other studies that used syndecan-1 to identify these cells in chronically inflamed synovia.^43^ ^44^

Syndecan-2 was present mainly in blood vessels where it occurred in endothelial cells, pericytes and smooth muscle cells. Expression of syndecan-2 has been shown in mesenchymal, neuronal and cancer cells.^45^ ^46^ In line with our results this HSPG has also been found in endothelial cells, for example in the brain where it is highly expressed.^47^ There is evidence that syndecan-2 is involved in angiogenesis that may be related to the interaction of the HSPG with vascular endothelial growth factor.^48^ This is of particular interest since in the RA synovium angiogenesis is a significant pathological event responsible for its enlargement and invasive properties, and suggests an involvement of syndecan-2 in these mechanisms.

Syndecan-3 was abundantly expressed in the endothelial cells of chronically inflamed synovia. This pattern resembled that of syndecan-2 but differed in being undetectable in the pericytes or smooth muscle cells of blood vessels. There has been a previous report of the expression of syndecan-3 by human endothelial cells. This has been shown in normal liver and intense reactivity occurs on endothelial cells in heptacellular carcinomas.^38^ ^49^ In addition, syndecan-3 has recently been shown in the blood vessels of non-malignant ovarian tissues with intense reactivity occurring on blood vessels in ovarian cancer.^50^ In the RA synovium, out of the syndecans and glypicans tested, syndecan-3 was the most abundantly detected HSPG in endothelial cells. Endothelial syndecan-3 selectively binds the chemokine CXCL8, suggesting a role in leukocyte trafficking into the synovium, whereas syndecan-2 and glypican-4 do not appear to bind this chemokine despite the presence of these HSPGs in the synovial endothelium.^14^ A role for syndecan-3 in leukocyte extravasation is further suggested since heparan sulphate can act as an adhesion molecule involved in blood leukocyte-endothelial interactions.^51^ In the current study syndecan-3 was not purely a marker of endothelial cells since it also localised to CD68+ macrophages in the sublining. In addition, the lining layer was positive for syndecan-3 suggesting that macrophages may also be positive in this layer, since macrophages are known to be a major component of the lining layer.^17^ In this connection macrophages have been shown to express syndecan-3 in rat liver^52^ and human liver with chronic cholestatic disease.^49^ Syndecan-3 is also expressed by chondrocytes in normal and OA articular cartilage where it is a regulator of chondrocyte proliferation.^12^ ^53^ Therefore, taken with our findings in the synovium, syndecan-3 appears to be an HSPG particularly associated with joint tissues.

Glypican-4 showed a similar distribution to that of syndecan-2, localising to endothelial cells, pericytes and smooth muscle cells in the walls of blood vessels. In addition, this HSPG occurred in the lining layer. By contrast, glypican-1 and glypican-3 were not detectable indicating that there is specificity in glypican expression in the synovium. Using the same antibody as ours, glypican-4 has been shown to be expressed in human kidney and bone marrow stromal and haematopoietic cells;^37^ ^54^ it is also expressed in development and binds fibroblast growth factor-2.^55^ ^56^

Several differences in HSPG expression were noted between inflamed and normal synovia. Syndecan-1 was abundantly expressed in RA and PsA samples whereas it was absent in normal and weakly expressed in OA. The lack of syndecan-1 in normal synovia relates to the absence of plasma cells in these samples whereas in RA and PsA these cells were abundantly present. Normal synovia were taken from patients with suspected meniscal damage of the knee and were essentially non-inflamed. The lack of lymphocytes of the B lineage is in agreement with a recent study by Singh *et al*^57^ who could not demonstrate L26+ B lymphocytes in normal knee synovia whereas CD3+, CD4+ and CD8+ T lymphocytes could be detected. Other changes in our study included an increased staining of syndecan-3 in endothelial cells, sublining macrophages and lining layer cells of RA and PsA in comparison to normal. Syndecan-2 staining was more intense in the blood vessels of RA and PsA compared with normal, and glypican-4 labelling was more intense in the lining layer of RA and PsA compared with normal. These results suggest that there is an upregulation of selected HSPGs in the chronically inflamed synovium.

The major difference in HSPG expression between the synovial samples appeared related to the degree of histological inflammation (tables 1 and 2). Inflammation, as characterised by the thickening of the synovial lining layer and infiltration of the sublining by leukocytes, was absent in normal synovia and increased in OA, RA and PsA. Similarly the level of HSPG expression was lowest in normal synovia and increased in OA, followed by RA and PsA. There was no obvious difference between the different types of chronic inflammation in terms of their HSPG expression, since RA and PsA showed an identical pattern. Therefore the difference in the pathologies of these two diseases, such as the respective presence or absence of rheumatoid factor (table 1), did not relate the presence or absence of particular syndecans or glypicans.

HSPGs act as co-receptors for cytokines and are involved in presenting chemokines to chemokine receptors.^1^ ^30^ ^58^ In addition, HSPGs act as adhesion molecules, playing an important role in cell–matrix and cell–cell interactions.^4^ ^29^ These functions are due to interactions with the heparan sulphate chains but may also include the core protein.^29^ ^42^ The role of the core protein of HSPGs is becoming increasingly apparent, not only determining when and where the heparan sulphate chains are expressed but also playing a direct role in signalling.^59^ Cell–matrix interactions have been shown for syndecan-3 and syndecan-1 that interact with extracellular molecules such as collagen and fibronectin.^4^ The presence of syndecan-3 on macrophages and syndecan-1 on plasma cells suggests that these HSPGs may be involved in leukocyte–matrix interactions leading to the accumulation of these cells in the inflamed synovial tissue. Furthermore, the presence of syndecan-2 and syndecan-3 on synovial endothelial cells could be involved in angiogenesis, chemokine presentation and leukocyte extravasation. With respect to angiogenesis, it is interesting that growth factors that are thought to drive this process in the RA synovium require binding to HSPGs for their activity, underlining an important potential role for HSPGs in this mechanism.^18^ ^60^ ^61^
